# Hollow carbon nanobubbles: monocrystalline MOF nanobubbles and their pyrolysis†
†Electronic supplementary information (ESI) available. See DOI: 10.1039/c6sc04903f



**DOI:** 10.1039/c6sc04903f

**Published:** 2017-03-07

**Authors:** Wei Zhang, Xiangfen Jiang, Yanyi Zhao, Arnau Carné-Sánchez, Victor Malgras, Jeonghun Kim, Jung Ho Kim, Shaobin Wang, Jian Liu, Ji-Sen Jiang, Yusuke Yamauchi, Ming Hu

**Affiliations:** a School of Physics and Materials Science , State Key Laboratory of Precision Spectroscopy , East China Normal University , Shanghai , 200241 , China . Email: mhu@phy.ecnu.edu.cn; b International Center for Materials Nanoarchitectonics (MANA) , National Institute for Materials Science (NIMS) , 1-1 Namiki , Tsukuba , 305-0044 , Japan; c Institute for Integrated Cell-Material Sciences , Kyoto University , Kyoto , 606-8501 , Japan; d Australian Institute for Innovative Materials (AIIM) , University of Wollongong , Squires Way , North Wollongong , NSW 2500 , Australia . Email: Yamauchi.Yusuke@nims.go.jp ; Email: yusuke@uow.edu.au; e Department of Chemical Engineering , Curtin University , Perth , WA 6845 , Australia . Email: jian.liu@curtin.edu.au

## Abstract

While bulk-sized metal–organic frameworks (MOFs) face limits to their utilization in various research fields such as energy storage applications, nanoarchitectonics is believed to be a possible solution. It is highly challenging to realize MOF nanobubbles with monocrystalline frameworks. By a spatially controlled etching approach, here, we can achieve the synthesis of zeolitic imidazolate framework (ZIF-8) nanobubbles with a uniform size of less than 100 nm. Interestingly, the ZIF-8 nanobubbles possess a monocrystalline nanoshell with a thickness of around 10 nm. Under optimal pyrolytic conditions, the ZIF-8 nanobubbles can be converted into hollow carbon nanobubbles while keeping their original shapes. The structure of the nanobubble enhances the fast Na^+^/K^+^ ion intercalation performance. Such remarkable improvement cannot be realized by conventional MOFs or their derived carbons.

## Abstract 2

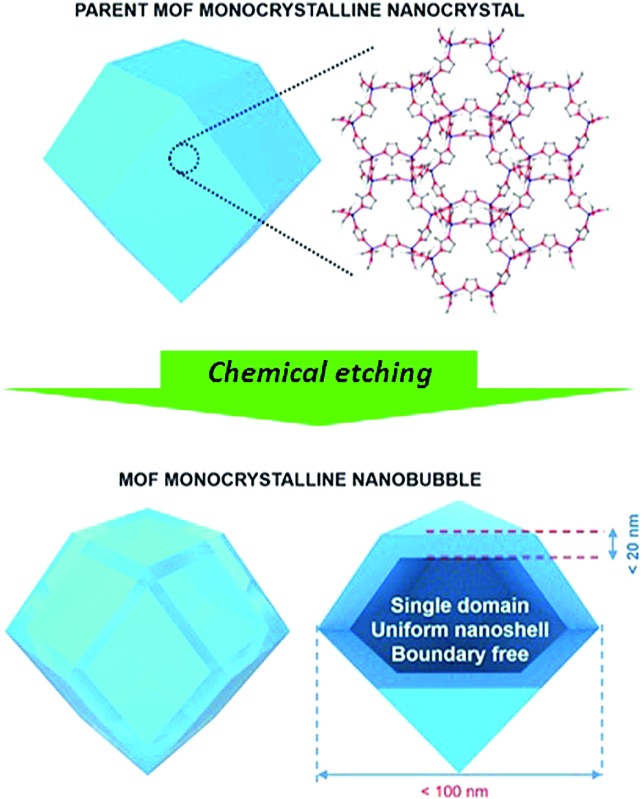
While bulk-sized metal–organic frameworks (MOFs) face limits to their utilization in various research fields such as energy storage applications, nanoarchitectonics is believed to be a possible solution.

## Introduction

Metal–organic frameworks (MOFs) and porous coordination polymers represent an emerging class of crystalline porous materials.^1–4^ While several interesting features, such as high surface area and uniform arrangement of nanopores, make MOFs attractive candidates for various applications, some limitations are still to be overcome for these materials to reach large-scale commercialization.^5–13^ When the size of a MOF crystal decreases below a critical diameter, size-dependent physicochemical properties become more significant (*e.g.*, fast sorption rates, unusual phase-transition behaviour).^14–17^

**Fig. 1:**
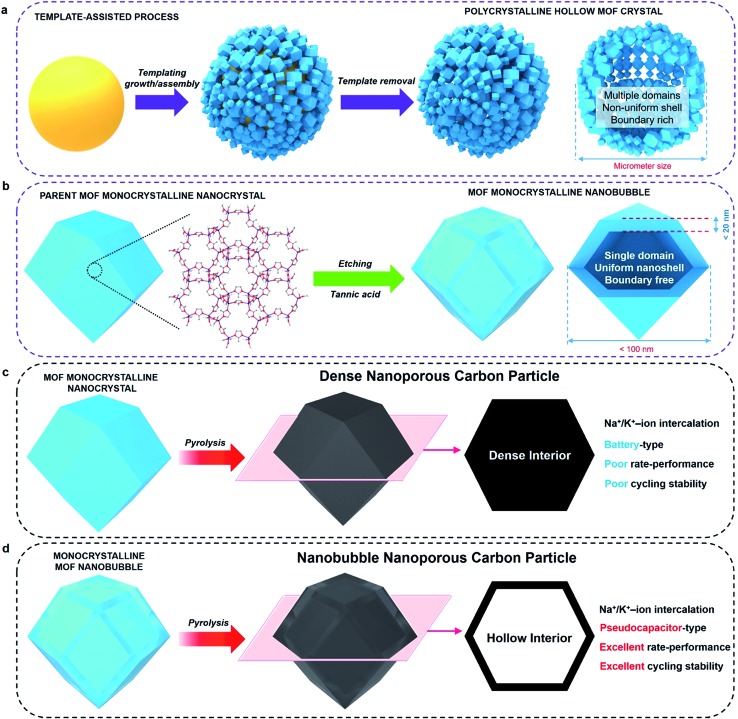
(a) Schematic illustration of the synthesis of hollow MOF crystals *via* traditional templating methods. (b) Schematic illustration of the spatially controlled etching to produce monocrystalline MOF nanobubbles. The nanobubbles are less than 100 nm in size, and have a monocrystalline shell with a thickness less than 20 nm. (c) Schematic illustration of the pyrolysis of MOF nanocrystals and description of Na^+^/K^+^ ion intercalation performance of the carbonized ZIF-8 nanocrystals (denoted as ‘non-hollow carbon nanoparticles’). (d) Schematic illustration of the pyrolysis of MOF nanobubbles and description of Na^+^/K^+^ ion intercalation performance of the carbonized MOF nanobubbles (denoted as ‘hollow carbon nanobubbles’).

Monocrystalline nanobubbles represent a special type of capsule-like nanostructure. The size of the nanobubbles can lead to small-scale effects. The nanoshells around the bubbles can provide anisotropic shape-dependent properties similar to those of nanofilms, nanosheets, or nanoflakes.^18^ The monocrystalline frameworks can guarantee a highly ordered crystallographic orientation, as well as a uniform thickness. Such nanostructures may be an ideal solution to improve the performance of MOFs and their derived materials in electrical energy storage (EES) applications. The synthesis of monocrystalline MOF nanobubbles is of great importance, and there have been several attempts towards synthesizing MOF capsules/nanobubbles.^19–26^ Most of them, however, were based on aggregation or self-assembly of MOF nanocrystals on soft or hard templates (Fig. 1a). Therefore, the obtained materials usually have polycrystalline frameworks or large particle size, which are not ideal when investigating the properties of such nanoarchitectures.

Here, we demonstrate the first synthesis of monocrystalline MOF nanobubbles through a controlled etching approach, which is different from the well-known templating methods, Kirkendall effect, or Ostwald ripening.^27–34^ Thanks to the development of etching techniques, the top-down fabrication of MOFs at a macro-size scale has been well-addressed recently.^31,34^ From these existing methods, we are now able to work at a nano-size scale. Protons can diffuse through the pores/channels inside the parent nanocrystals, thus leading etching the core region with nanoscale precision while preserving the monocrystalline framework of the outer region (Fig. 1b). The obtained MOF nanobubbles are of a small size, and they feature thin shells and monocrystalline frameworks, which allows us to explore their nanobubble-correlated physicochemical properties and extend their range of application. The mother MOF nanoparticle and MOF nanobubbles were pyrolyzed to convert into nanoporous carbons (Fig. 1c and d). The carbonaceous nanobubbles possess the structure that can change the ultra-fast Na^+^/K^+^ ion intercalation from battery-type to pseudocapacitor-type.^35^

## Experimental

### Synthesis of hollow ZIF-8 nanobubbles and hollow carbon nanobubbles

Zn(NO_3_)_2_ (258 mg) and 2-methylimidazole (263 mg) were first mixed together. Then, methanol (40 mL) was added to dissolve the mixed powders. The solution was stirred for 10 min, followed by incubation at room temperature for 24 hours. The resultant white precipitate (ZIF-8 nanocrystals) was obtained, washed with methanol, and dried for further use. 2 mg of the as-prepared ZIF-8 nanocrystals were incubated in 1 mL of a tannic acid solution (5 g L^–1^) and aged for 5 min. The hollow ZIF-8 nanobubbles were collected by centrifugation and washed with water and methanol. For pyrolysis of the ZIF-8 powders, 100 mg of the hollow ZIF-8 nanobubbles were heated at 600 °C for 5 h under N_2_ atmosphere. The collected black powder was then washed with concentrated HCl solution to completely remove the residual Zn or ZnO. After washing with ethanol and water repeatedly, the obtained black sample was finally dried under vacuum at 100 °C.

### Characterization

Transmission electron microscope (TEM) observations were performed using a JEOL 2100F TEM system operated at 200 kV. The morphologies were observed by a Hitachi SU-8000 field emission scanning electron microscope (SEM) at an accelerating voltage of 5 kV. Wide-angle powder X-ray diffraction (PXRD) patterns were obtained with a Rigaku Smartlab diffractometer with monochromated Cu Kα radiation (40 kV, 40 mA) at a scanning rate of 1° min^–1^. Nitrogen adsorption–desorption data were obtained with an Autosorb IQ Gas Sorption System at 77 K. Zeta-potential measurements were performed with a Malvern Zetasizer Nano ZS. Fourier transform infrared (FTIR) spectra of the samples were obtained with a FTIR spectrophotometer (Varian 7000).

### Electrochemical measurements

All the electrochemical measurements were carried out using coin cells (CR 2032). The working electrode was fabricated by mixing 80% as-prepared materials, 10% carbon black, and 10% poly(vinylidene difluoride) (PVDF) in *N*-methyl-2-pyrrolidone (NMP). The slurry was coated onto Cu foil and dried at 100 °C for 15 h in a vacuum oven. The mass loading was 2 mg cm^–2^. Glass fibres (GF/D) from Whatman were utilized as separators. For the sodium ion batteries, sodium metal was used as both counter electrode and reference electrode. The electrolyte used was 1 M NaClO_4_ dissolved in ethylene carbonate (EC) and propylene carbonate (PC) (1 : 1 by volume) with 5% fluorinated ethylene carbonate as an electrolyte additive. For the potassium ion batteries, potassium metal was used as both counter electrode and reference electrode. The electrolyte used was 0.8 M KPF_6_ dissolved in ethylene carbonate (EC) and diethyl carbonate (DEC) (1 : 1 in volume). All the cells were assembled in an argon-filled glove box with moisture and oxygen levels below 0.1 ppm. The galvanostatic charge–discharge testing was performed on a Land CT 2001A battery test system (Wuhan, China). The cyclic voltammetry (CV) measurements were performed on a CHI electrochemical workstation (CHI, 660E).

## Results and discussion

### Synthesis of the monocrystalline MOF nanobubbles

**Fig. 2:**
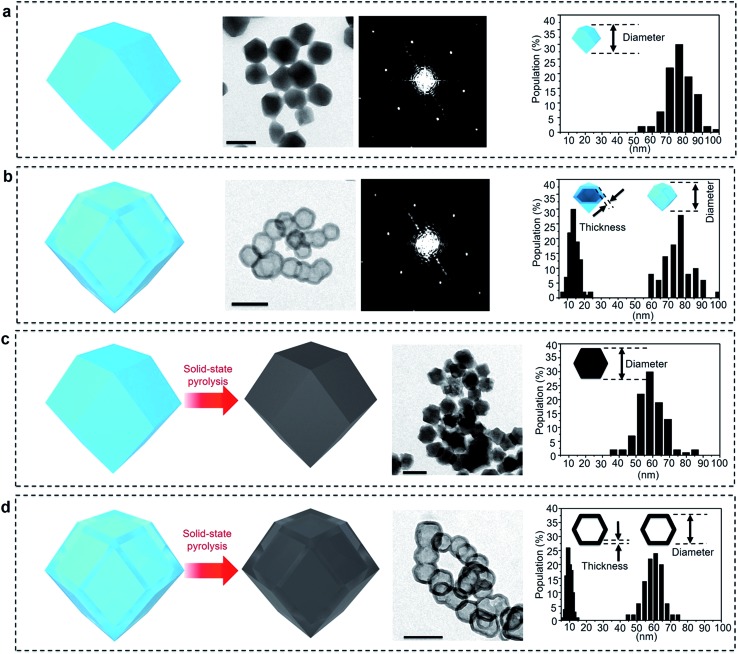
From left to right, illustration, TEM image, SAED pattern, and particle size distribution (with shell thickness for nanobubbles) of the (a) parent ZIF-8 nanocrystals, (b) ZIF-8 nanobubbles after etching, (c) carbonized ZIF-8 nanocrystals (non-hollow carbon nanoparticles), and (d) carbonized ZIF-8 nanobubbles (hollow carbon nanobubbles). The scale bars in all the images correspond to 100 nm. The SAED patterns were collected from individual nanocrystals/nanobubbles. The size–distribution profiles were analysed by counting 100 nanocrystals/nanobubbles.

Zeolitic imidazolate frameworks-8 (ZIF-8) was selected as a typical MOF.^36^ Fig. 2a and S1a† present TEM and SEM images of the parent ZIF-8 nanocrystals, respectively. The nanoparticles have a rhombic dodecahedral structure with truncated corners. The average particle size was measured for over 100 particles and was found to be around 75 nm. PXRD pattern of the nanoparticles matches the simulated pattern of pure sodalite-type ZIF-8 single-crystal data (Fig. S2†). The selected area electron diffraction (SAED) pattern taken from one single particle reveals periodic spots, which can be assigned to a single-crystal structure (Fig. 2a).

After simply immersing the parent ZIF-8 nanocrystals in a tannic acid (C_76_H_52_O_46_) solution for 5 min, the centre of the particles was adequately removed, as shown in Fig. 2b and S1b,† indicating the occurrence of internal etching. Nanobubbles with thin shells around 15 nm thick were obtained without any by-products, such as irregularly etched particles. The shape and sizes of the etched sample particles are similar to the ones from the initial parent ZIF-8 (Fig. S1b†). Although other hollow MOF crystals have been reported previously,^19–26^ the shells commonly have a rough surface composed of aggregated nanoparticles, as shown in Fig. 1a. In our case, the surface of the shell is smooth and retains the parent monocrystalline nature, as confirmed by SAED patterns (Fig. 2b). The PXRD pattern also confirms that the obtained nanobubbles possess the same crystal structure as the initial ZIF-8 nanoparticles (Fig. S2†). These results clearly suggest that monocrystalline ZIF-8 nanobubbles can be obtained *via* a chemical etching approach. This method is applicable to other metal-imidazole coordinated MOFs (with the case of ZIF-7 shown in Fig. S3†).

To understand the interaction between tannic acid and ZIF-8, an *in situ* pH change of the suspension of ZIF-8 was monitored (Fig. S4†). The pH value of the tannic acid solution is as low as 3.8, suggesting that protons can be released from tannic acid. After adding ZIF-8 nanoparticles, the pH value was increased to 6.1 immediately, indicating that the protons were captured by ZIF-8. The etching of ZIF-8 probably happens at this stage. Then, the pH value gradually increased to 8 within 10 min. This is because of the 2-methylimidazole released from ZIF-8 which can neutralize the protons. As the pH becomes basic, the etching of ZIF-8 stops automatically. Considering the short etching duration, ZIF-8 can only be dissolved partly. To clarify why the dissolved part is mainly in the centre rather than at the surface, the surface zeta-potentials of the non-hollow ZIF-8 and hollow ZIF-8 were compared. The initial zeta potential of the ZIF-8 is positive (+20 mV). After etching, the surface of the particles change from positive to negative (–38 mV), indicating that tannic acid is adsorbed. The adsorption of tannic acid is also supported by FTIR (Fig. S5†). Vibration and stretching bands of tannic acid are present in the spectrum recorded from the etched ZIF-8. The bands marked by arrows come from the adsorbed tannic acid.^37^

To see whether polymeric acids other than tannic acid can be employed to fabricate hollow ZIFs, polymaleic acid and hyaluronic acid were employed. In the presence of polymaleic acid, ZIF-8 particles are etched but the shapes are not well-defined (Fig. S6†). The shells are partly destroyed, because the etching cannot be confined in the centre of ZIF-8. The reason is probably that the polymaleic acid cannot coat on the surface of ZIF-8 uniformly owing to the linear conformation of the molecule and the scarcity of hydroxyl groups. In the case of hyaluronic acid, the ZIF-8 nanoparticles cannot be etched at all (Fig. S7†). The reason is that hyaluronic acid cannot provide enough protons to induce disassociation of ZIF-8.

To demonstrate whether the stability of MOFs against protonation is a critical factor, we further applied this tannic acid etching method for other MOFs such as MIL-101 or UIO-66. Both MIL-101 and UIO-66 are stable in acid solution. After etching, the nanocrystals remain unaffected (Fig. S8 and S9†). The absence of internal cavity confirms that the weak stability of MOFs is critical for this technique to be successful.

In other words, tannic acid acts as a protecting agent as well as an etching agent, while ZIF-8 provides the necessary triggers to interrupt the etching automatically. Tannic acid releases protons (p*K*_a_ = 10)^37^ to penetrate the surface and reach the central region of the ZIFs through the interconnected pores/channels to dissolve the core. In the meantime, the planar conformation and abundance of hydroxyl group allow the molecules to be adsorbed on the surfaces of the ZIF nanoparticles uniformly. Since tannic acid is too large to penetrate the MOFs, the adsorbed molecules can cover most of the surface, thus protecting the outer region of the crystals. The fast etching takes place inside the nanocrystals, thereby leading to monocrystalline MOF nanobubbles. The released organic ligand from ZIF-8 raises the pH and stops the etching before reaching complete dissolution. Such cooperation between ZIF-8 and tannic acid is crucial for obtaining a precise and uniform etching.

### Conversion of ZIF-8 nanobubbles into hollow carbon nanobubbles

To evaluate the correlation between the nanostructure and the electrochemical properties of the ZIF-8 nanobubbles, the solid-state conversion of ZIFs can be modelled. Solid-state pyrolysis of MOFs has been recently recognized as critical for their applications.^11–14^ Compared materials prepared from other sources, solids derived from ZIFs show attractive properties, such as high porosity and electrochemical activity.^7–10^ Here, nanoporous carbons are prepared by pyrolyzing the parent ZIF-8 nanocrystals and nanobubbles, as illustrated in Fig. 1c and d and 2c and d.

The ZIF-8 nanocrystals and nanobubbles are carbonized under the same conditions (at 600 °C under N_2_ atmosphere), followed by an extensive etching of the remaining Zn species with a HCl solution. Elemental analysis confirms that both the samples are converted into carbonaceous materials. The products are mainly composed of carbon and nitrogen, while hydrogen is present as well. Fig. 2c and d show the TEM images of both samples after pyrolysis. Both samples retain a similar morphology to the initial ZIF-8 nanocrystals and nanobubbles. Hereafter, the carbons derived from the parent ZIF-8 nanocrystals are denoted as “non-hollow carbon nanoparticles”, while the carbons derived from the ZIF-8 nanobubbles are denoted as “hollow carbon nanobubbles”. The particle size and shell thickness distributions are shown in the rightmost column. The average size of both non-hollow carbon nanoparticles and hollow carbon nanobubbles is around 60 nm (with a shell thickness of around 10 nm for the nanobubbles). The thickness and particle size are slightly reduced compared to the parent ZIF samples, because of the removal of Zn and carbonization of the organic components.

**Fig. 3:**
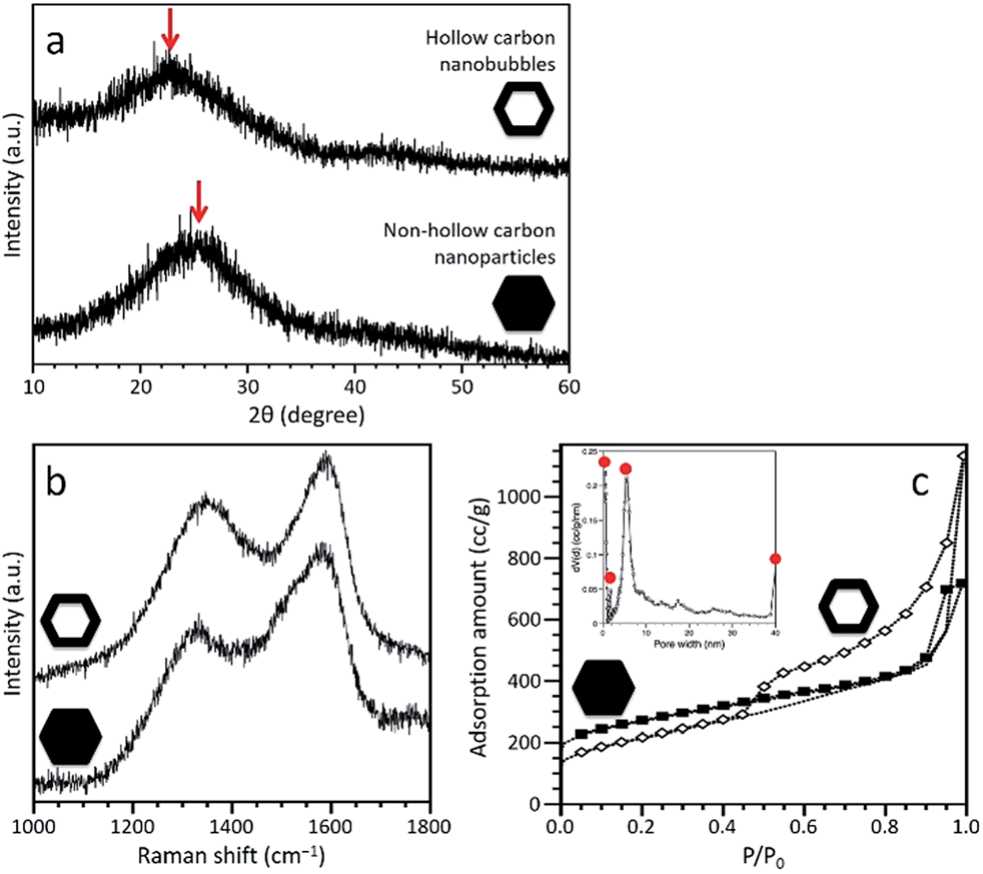
(a) Wide-angle PXRD patterns, (b) Raman spectra, and (c) N_2_ adsorption–desorption isotherms of the non-hollow carbon nanoparticles and hollow carbon nanobubbles. The pore size distribution of the hollow carbon nanobubbles is calculated by the non-local density functional theory (NLDFT) method, as shown in the inset of (c).

PXRD profiles of both carbonaceous samples are shown in Fig. 3a, confirming that both samples are amorphous, although partly graphitized. A broad peak is centred at 24.1° for the non-hollow carbon nanoparticles and at 21.8° for the hollow carbon nanobubbles, as shown by the arrows in Fig. 3a. Each peak come from the graphitic domain of their respective sample. This shift suggests that the interlayer spacing of the graphitized layers in both samples is different. According to the (002) peak, the average interlayer distance increased from 0.35 nm for the nanoparticles to 0.41 nm for the nanobubbles. The Raman spectra of both samples exhibit broad disorder-induced D-band and in-plane vibrational G-band (Fig. 3b). The integral intensity ratio (*I*_G_/*I*_D_) of the hollow carbon nanobubbles is 1.06, which is smaller than the value for the non-hollow carbon nanoparticles (1.13). The lower value of *I*_G_/*I*_D_ for the nanobubbles suggests a lower degree of graphitization, which matches their larger interlayer spacing.^38^ There are several possible reactions that occur during the carbonization process, such as the generation of gases, the movement of carbon, nitrogen, and hydrogen atoms, and the re-formation of graphitic structure. The mobility of carbon atoms inside the monocrystalline ZIF-8 nanobubbles during pyrolysis is probably limited by the confinement in two-dimensional space. Therefore, the degree of graphitization for the hollow carbon nanobubbles is lower.

N_2_ adsorption–desorption isotherms were collected to compare the porosity of both samples (Fig. 3c). A large increase at low relative pressure (*P*/*P*_0_ < 0.1) indicates the presence of micropores. A clear hysteresis loop is observed for the hollow carbon nanobubbles, suggesting the presence of a significant amount of randomly arranged mesopores. The specific surface areas are ∼900 m^2^ g^–1^ for the non-hollow carbon nanoparticles and ∼700 m^2^ g^–1^ for the hollow carbon nanobubbles. The pore size distribution for the hollow carbon nanobubbles shows micropores (less than 2 nm), small-sized mesopores (∼6 nm), and large-sized mesopores (*i.e.*, hollow cavity size, ∼40 nm), as shown in the inset of Fig. 3c. Small-sized mesopores are probably formed due to the diffusion of protons during the etching process.

### Electrochemical performance

Nanoporous carbons have been widely utilized in various applications.^39–41^ Recently, they have also been recognized as promising anode materials for sodium ion batteries, which are considered as a potential alternative to the lithium ion battery because of the global abundance and low cost of their main components.^42–45^ To evaluate the electrochemical performance of the hollow carbon nanobubbles, half-cell tests of sodium ion battery were carried out.

**Fig. 4:**
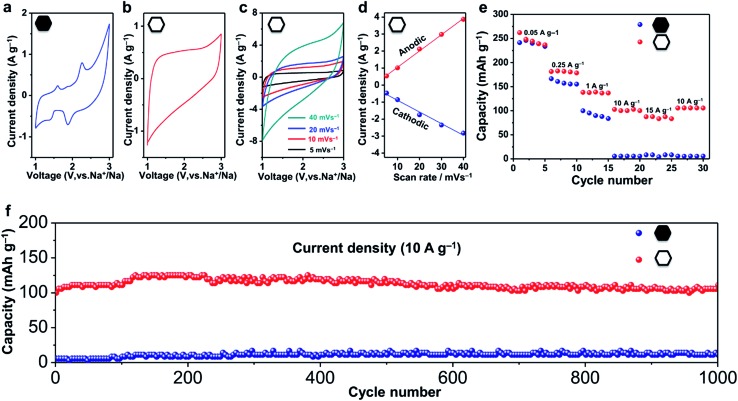
CV curves of (a) the non-hollow carbon nanoparticles and (b) hollow carbon nanobubbles between 1 and 3 V (*vs.* Na^+^/Na) at a potential sweeping rate of 5 mV s^–1^. (c) CV curves of the hollow carbon nanobubbles between 1 and 3 V (*vs.* Na^+^/Na) at various rate in the range of 5–40 mV s^–1^. (d) The dependence of anodic and cathodic current (at 2 V) on scanning rate. (e) Rate performance and (f) cycling performance of the hollow carbon nanobubbles and non-hollow carbon nanoparticles. The electrodes were firstly activated by charging/discharging at a current density of 50 mA g^–1^ before testing at the current density of 10 000 mA g^–1^, then tested between 0.001–3 V (*vs.* Na^+^/Na).

The shape of the CV curves and the kinetics of the ion storage are two characteristics which are necessary to assess to distinguish the intercalation behavior.^35,46^ A battery-type intercalation usually results in a CV curve with significant faradaic redox peaks, while a pseudocapacitor-type intercalation has a quasi-rectangular curve. The kinetics during the ion intercalation can be described as *i* = *Cv* ^*b*^, where *i* is the current (A), *C* and *b* are adjustable values, and *v* is the sweep rate (mV s^–1^).^46^ In the case of battery-type intercalation, the kinetics is hyperbolic (*b* < 1), while for the pseudocapacitor-type intercalation, the kinetics is liner (*b* = 1). The pseudocapacitor-type ion storage can allow a faster insertion/extraction of ions than the battery-type intercalation, and can thus benefit from fast electrochemical energy storage. Herein, we have investigated the shape of the CV curves and added the kinetic information obtained from sweep voltammetry of the hollow and non-hollow materials (Fig. 4a–d and S10†).

The CV curves of the non-hollow carbon nanoparticles are shown in Fig. 4a and S10.† Redox peaks can be observed in the range of 1.5–3.0 V and 0.0–0.5 V. In contrast, the CV curves of the hollow carbon nanobubbles exhibit a stable quasi-rectangular-shape (Fig. 4b).^46^ The ion storage kinetics of both materials are also distinct. The kinetics of the hollow carbon nanobubbles and of the non-hollow carbon nanoparticle can be fitted with *i* = *Cv* and *i* = *Cv* ^0.3^, respectively. All these results suggests that the hollow carbon nanobubble have a pseudo-capacitor-type ion storage, while the non-hollow carbon nanoparticles seem to adopt a battery-type ion intercalation.

The capacitor-type intercalation behaviour of the hollow carbon nanobubbles is interesting because it offers high levels of charge storage within a short period.^46^ Herein, enhanced rate-performance is achieved by using the hollow carbon nanobubbles as a negative electrode. Charge/discharge profiles of the hollow carbon nanobubbles measured between 0.001 and 3.000 V *versus* Na^+^/Na at 50 mA g^–1^ are shown in Fig. S11.† The first charge and discharge exhibit specific capacities of 260 and 380 mA h g^–1^, respectively. At a low current density (50 mA g^–1^), the hollow carbon nanobubbles and non-hollow carbon nanoparticles almost deliver the same capacity (about 240 mA h g^–1^, see Fig. 4e). When the current density is increased up to 15 000 mA g^–1^, however, the capacity of the hollow and non-hollow structures drop down to 90 mA h g^–1^ and 4 mA h g^–1^, respectively, thus showcasing the superior charge storage ability of the nanobubbles.

The hollow carbon nanobubbles also show superior cycling stability at a high current density (10 000 mA g^–1^) (Fig. 4f). The electrode containing the hollow carbon nanobubbles delivers a discharge capacity of 100 mA h g^–1^ in the first cycle, and remains unchanged after 1000 cycles. The coulombic efficiency approaches 99% over 1000 cycles (Fig. S12†). The samples were observed by TEM after 1000 charge/discharge cycles (Fig. S13†). Clearly, both the hollow and non-hollow carbons are stable during the electrochemical testing. Therefore, the significant differences in the rate-performance and cycling stability between the two samples are not caused by differences in the stability of the materials, but can be explained by the correlation between the sodium storage behaviour and the nanostructure.

**Fig. 5:**
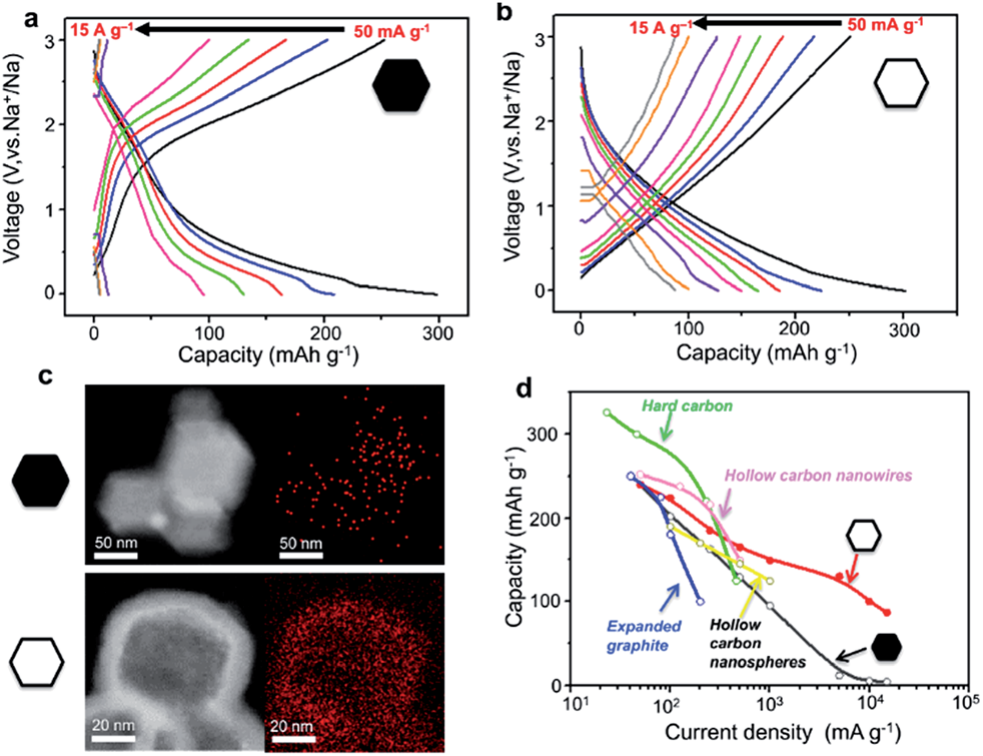
Galvanostatic charge/discharge curves of (a) the non-hollow carbon nanoparticles and (b) the hollow carbon nanobubbles between 0.001 and 3.000 V (*vs.* Na^+^/Na) at various current densities, ranging from 50 to 15 000 mA g^–1^. (c) Elemental mapping of sodium content in the non-hollow nanoparticles and the hollow nanobubbles after insertion of Na^+^ ions at a current density of 10 000 mA g^–1^. The red pixels represent sodium. (d) Rate capability for hard carbon,^42^ expanded graphite,^11^ hollow carbon nanowires,^41^ hollow carbon nanospheres,^44^ and our non-hollow carbon nanoparticles and hollow carbon nanobubbles.

To highlight the correlation between the high-rate performance and the sodium storage behaviour of the hollow carbon nanobubbles, the charge/discharge profiles of the hollow carbon nanobubbles and non-hollow carbon nanoparticles at various current densities are shown in Fig. 5a and b. The hollow carbon nanobubbles can maintain their capacitor-type behaviour upon increasing the current density from 50 to 15 000 mA g^–1^. The non-hollow carbon nanoparticles show typical battery-type behaviour, which is normal for the intercalation of Na^+^ ions. The pseudocapacitor intercalation behaviour is the reason for such high-rate performance in the hollow carbon nanobubbles. The elemental mapping of sodium suggests that a high density of Na^+^ ions penetrates uniformly the entire hollow carbon nanobubble structure, even at current density as high as 10 000 mA g^–1^, while only few ions can be found in non-hollow carbon nanoparticles after insertion (Fig. 5c). This is a direct evidence of the achievement of high capacity retention at current densities higher than 1000 mA g^–1^ (Fig. 5d). Compared with other carbons (hard carbon,^43^ expanded graphite,^15^ hollow nanowires,^42^ hollow nanospheres^45^), our hollow carbon nanobubbles show competitive and promisingly high capacity.

Analogically, potassium ion batteries are also known for being a sustainable alternative to lithium ion batteries. Although K^+^ ions are bigger than Na^+^ ions, their stronger interaction with graphitic carbon can be a motivating factor for combining them with carbon-based anodes.^11–13,47^ Our hollow carbon nanobubbles show promising performance when implemented in such devices (see Fig. S14 and S15†). To demonstrate the potential of the hollow carbon nanobubbles for K^+^ ion insertion/extraction, half-cell tests are performed. Fig. S14a† shows the CV curves of the hollow carbon nanobubbles and the non-hollow nanoparticles. The formation of SEI films can be deduced from the appearance of an irreversible peak at 0.75 V for both samples. After the first cycle of intercalation, the CV curve of the hollow carbon nanobubbles presents a quasi-rectangular shape, corresponding to pseudocapacitor behaviour. In contrast, the CV curve of the non-hollow carbon nanoparticles has a broad redox peak in the voltage range from 2 to 3 V. Charge/discharge profiles measured between 0.001 and 3.000 V *vs.* K/K^+^ at 50 mA g^–1^ are shown in Fig. S14b.† The first charge capacity of the hollow carbon nanobubbles (330 mA h g^–1^) is significantly larger than the capacity of the nanoparticles (200 mA h g^–1^), thus highlighting their stronger interactions with K^+^ ions.

The rate performance of the hollow carbon nanobubbles is also higher than that of the non-hollow carbon nanoparticles, similarly to the Na^+^ ion battery tests. When the current density increased up to 2 A g^–1^, the retained capacity of the hollow carbon nanobubbles is 100 mA h g^–1^, while the non-hollow carbon nanoparticles could only deliver a capacity of around 20 mA h g^–1^ (Fig. S14c†). The good cycling stability of the hollow carbon nanobubbles at a high current density (1000 mA g^–1^) is shown in Fig. S14d.† The electrode containing the hollow carbon nanobubbles delivered a capacity of 130 mA h g^–1^ in the first cycle and retained a reversible capacity of 100 mA h g^–1^ after 50 cycles. The non-hollow carbon nanoparticles showed a capacity of 25 mA h g^–1^ in the first cycle and a retained capacity of 20 mA h g^–1^ after 50 cycles.

The promising rate-performance and cycling stability of the hollow carbon nanobubbles can be attributed to their capacitor-type behaviour as demonstrated by the charge/discharge profiles at various current densities. The curves of the hollow carbon nanobubbles retain their capacitor-type shape when the current density is increased from 50 up to 2000 mA g^–1^ (Fig. S15a and b†). The curves of the non-hollow carbon nanoparticles have a plateau, suggesting a typical battery-type behaviour, which is normal for the intercalation of K^+^ ions. The pseudocapacitor K^+^ ion intercalation behaviour of the nanobubbles is similar to their Na^+^ ion intercalation behaviour, which demonstrates that the hollow nanostructure facilitate fast ion storage. This property is responsible for their good rate-capacity compared to previously reported carbon materials (Fig. S15c†).

We further utilized the hollow and non-hollow carbon nanoparticles as host for sulphur in Li–S battery. The electrode using hollow carbon nanobubbles shows an improved cycling stability than the electrode made by non-hollow nanoparticles (Fig. S16†). Such a result suggests that the hollow structure has the ability to enhance the performance of nanoporous carbons for Li–S battery.

## Conclusions

Here, we have reported a spatially controlled etching strategy to prepare monocrystalline ZIF nanobubbles. The nanoetching strategy can be extended to many other MOFs, with various size or shape and with interconnected pores/channels, respective to the parent material. The direct carbonization of MOF nanobubbles is used as a simple and flexible pathway to fabricate hollow carbon nanobubbles. With traditional approaches, the shapes and structures of the hollow MOFs are limited. In contrast to the previous methods, the present work represents a promising strategy to fabricate nanosize monocrystalline hollow MOF nanobubbles. Our hollow carbon nanobubbles have significant advantages for EES applications. Although several approaches have been reported for hollow nanoporous carbons, such uniformlysized hollow nanobubbles with thin shells have never been reported. The commonly employed templating methods are complicated and usually involve template functionalization (*e.g.*, silica spheres), deposition of precursors, carbonization, template removal, and activation of the obtained carbons. In our case, the hollow carbon nanobubbles can be easily prepared through carbonization of the hollow MOF nanobubbles. Interestingly, the nanobubble structure sets the Na^+^/K^+^ ion intercalation in MOF-derived carbons to a pseudocapacitor-type. Ion intercalation is slow in most batteries, but fast ion storage does occur through surface redox reactions or ultrafast ion intercalation in pseudocapacitors.^46^ The nanobubbles not only have a nanosize diameter (<70 nm), but also have a thin shell structure (around 10 nm in thickness), promoting a shorter diffusion pathway and more easily accessible surfaces for ions than the corresponding non-hollow nanoparticles. In this work, the intercalation of the Na^+^/K^+^ ions in the nanobubbles can be fast, leading to a capacitor-type intercalation behaviour. These results demonstrate that nanoshell-dependent electrochemical properties constitute an exciting alternative to achieve superior battery performance.
