# Supplementary material for: Hollow carbon nanobubbles: monocrystalline MOF nanobubbles and their pyrolysis
Source: Chem Sci. 2017 Mar 7;8(5):3538–46. doi: 10.1039/c6sc04903f (PMC5436298; doi:10.1039/c6sc04903f)
Supplement: Supplementary file 1 [file SC-0008-C6SC04903F-s001.pdf]

Electronic Supplementary Material (ESI) for Chemical Science.
This journal is © The Royal Society of Chemistry 2017

Electronic Supporting Information

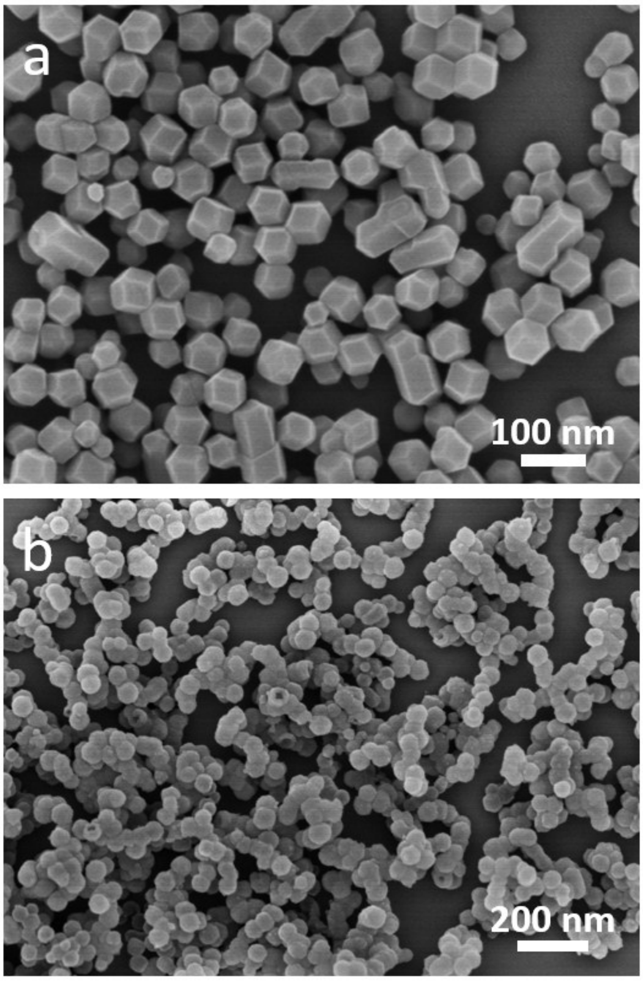

Fig. S1 SEM images of (a) the parent ZIF-8 nanocrystals and (b) the etched ZIF-8 nanobubbles.

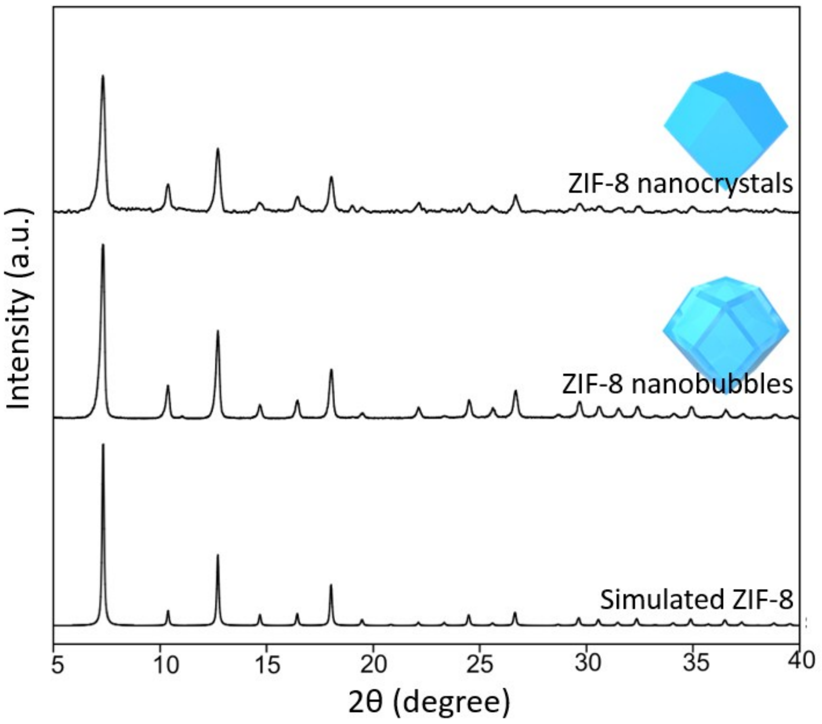

Fig. S2 Wide-angle PXRD profiles of the parent ZIF-8 nanocrystals, the ZIF-8 nanobubbles, and the simulated ZIF-8 pattern from single-crystal data.

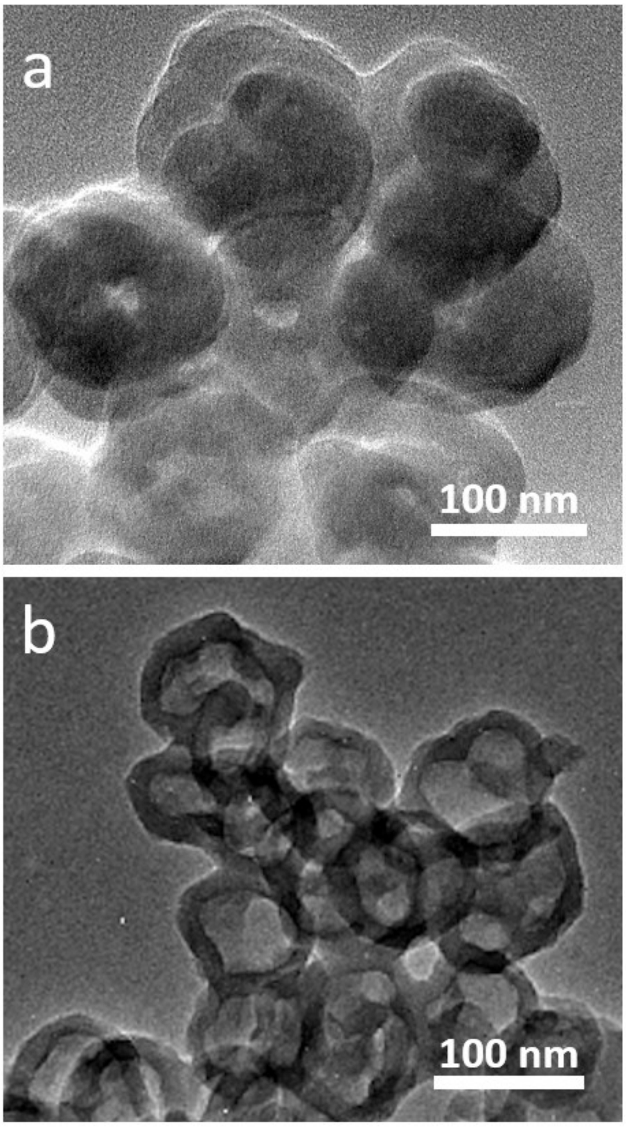

Fig. S3 TEM images of (a) the parent ZIF-7 nanocrystals and (b) the etched ZIF-7 nanobubbles.

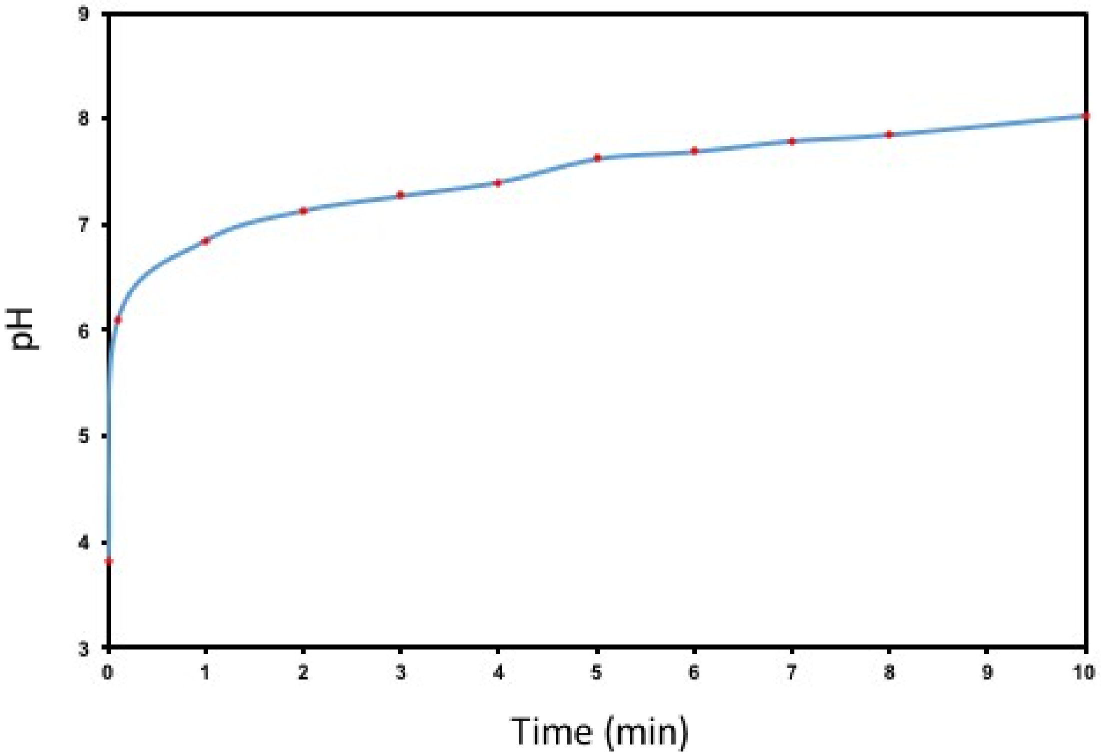

Fig. S4 pH change of the suspension of ZIF-8 nanoparticles in tannic acid versus time.

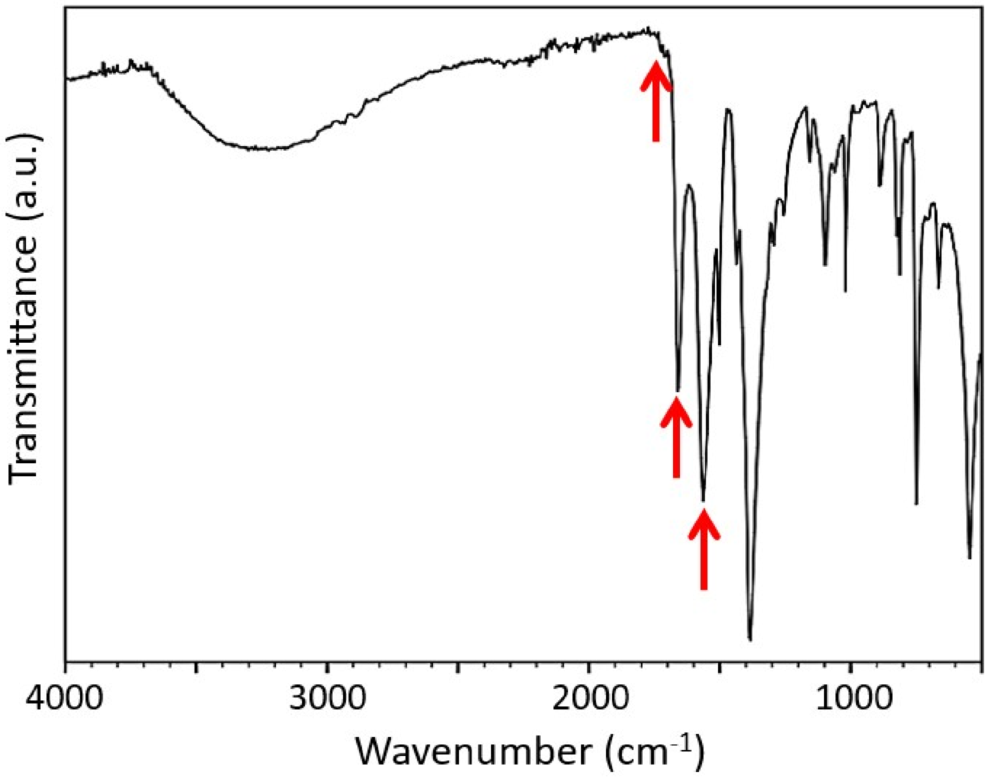

Fig. S5 FTIR spectrum of the ZIF-8 nanobubbles treated with tannic acid molecules. The arrows indicate the typical bands that are assignable to the adsorbed tannic acid molecules.

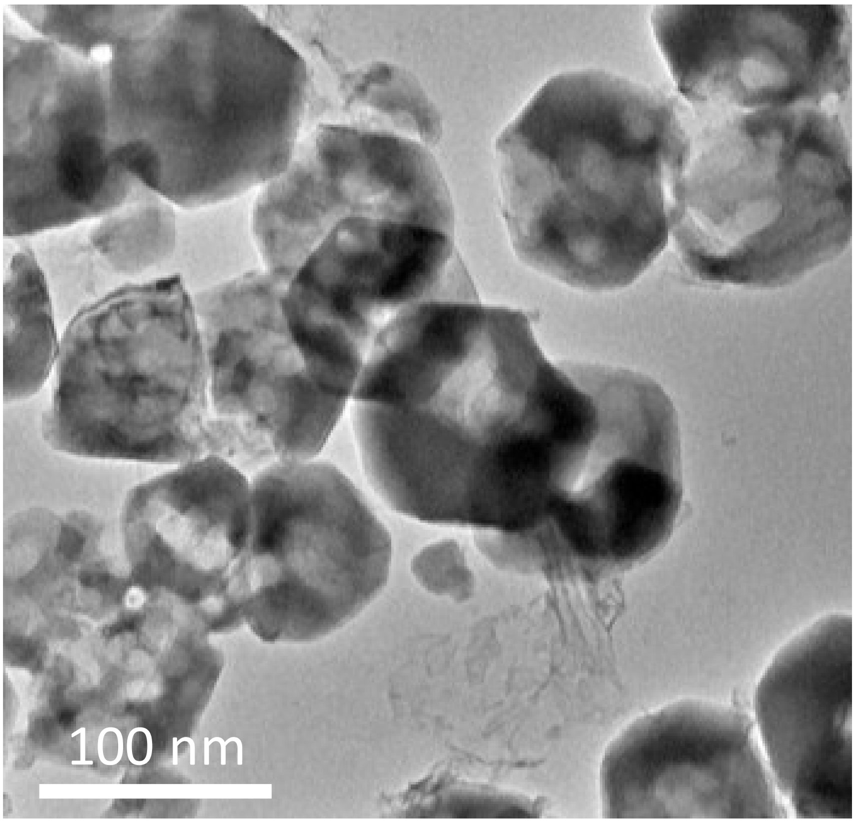

Fig. S6 TEM images of ZIF-8 etched by polymaleic acid.

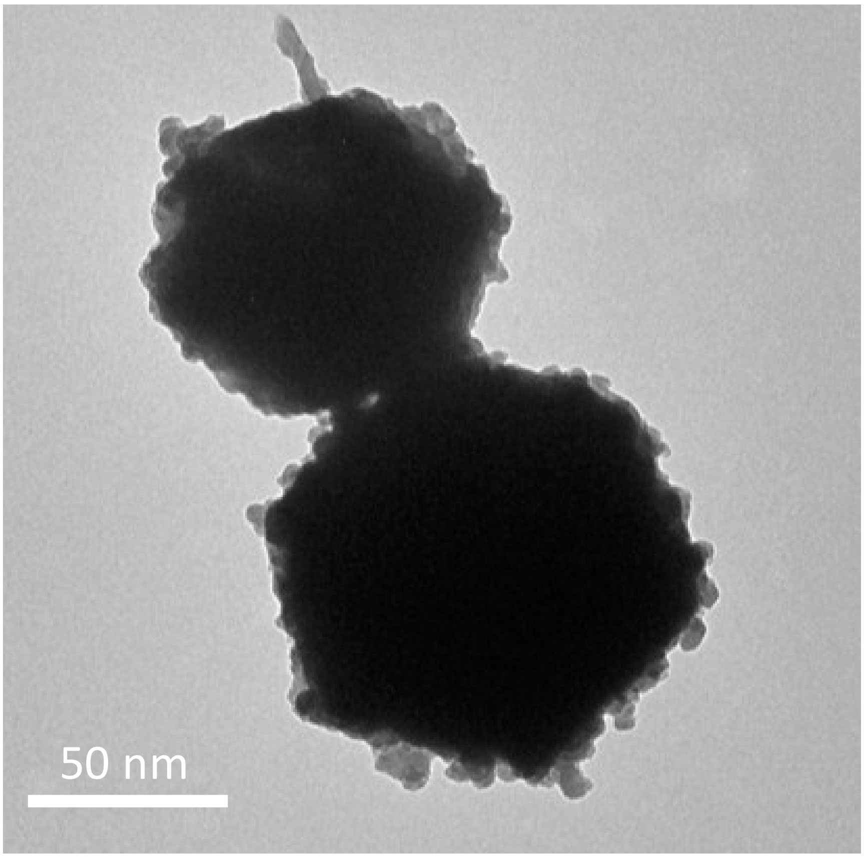

Fig. S7 TEM images of ZIF-8 etched by hyaluronic acid.

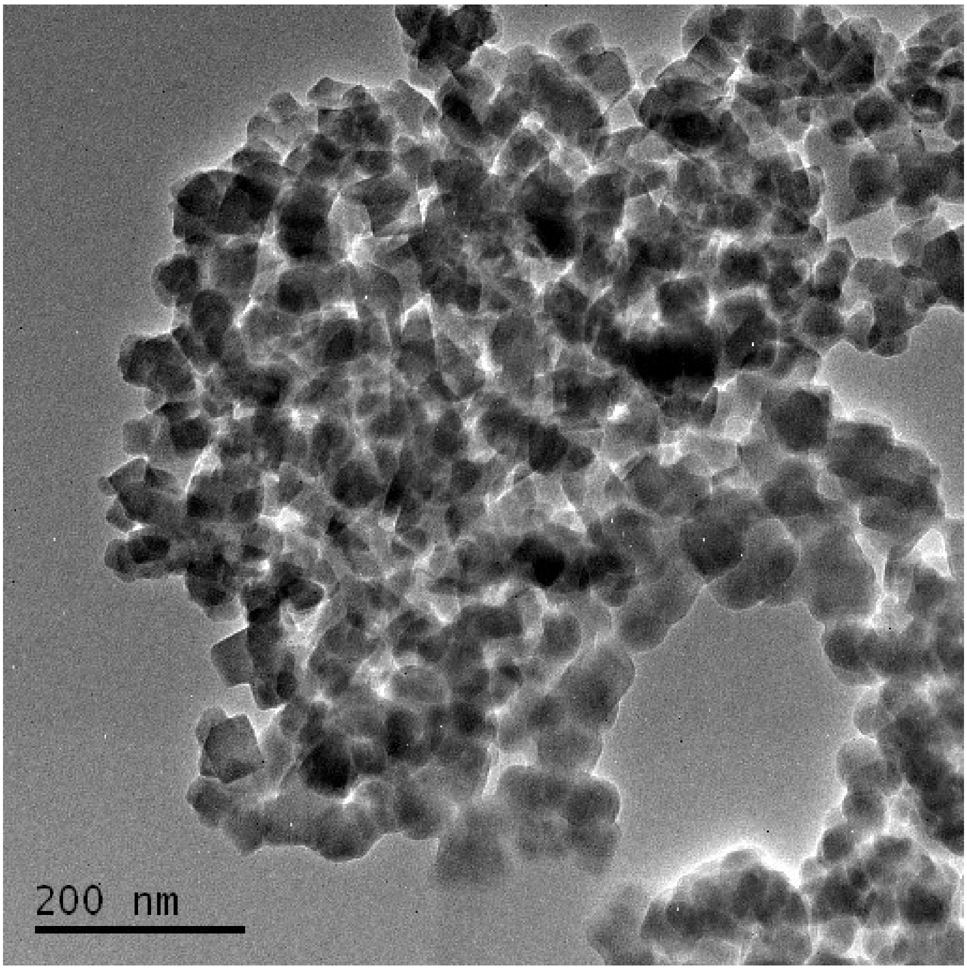

Fig. S8 TEM images of UIO-66 etched by tannic acid.

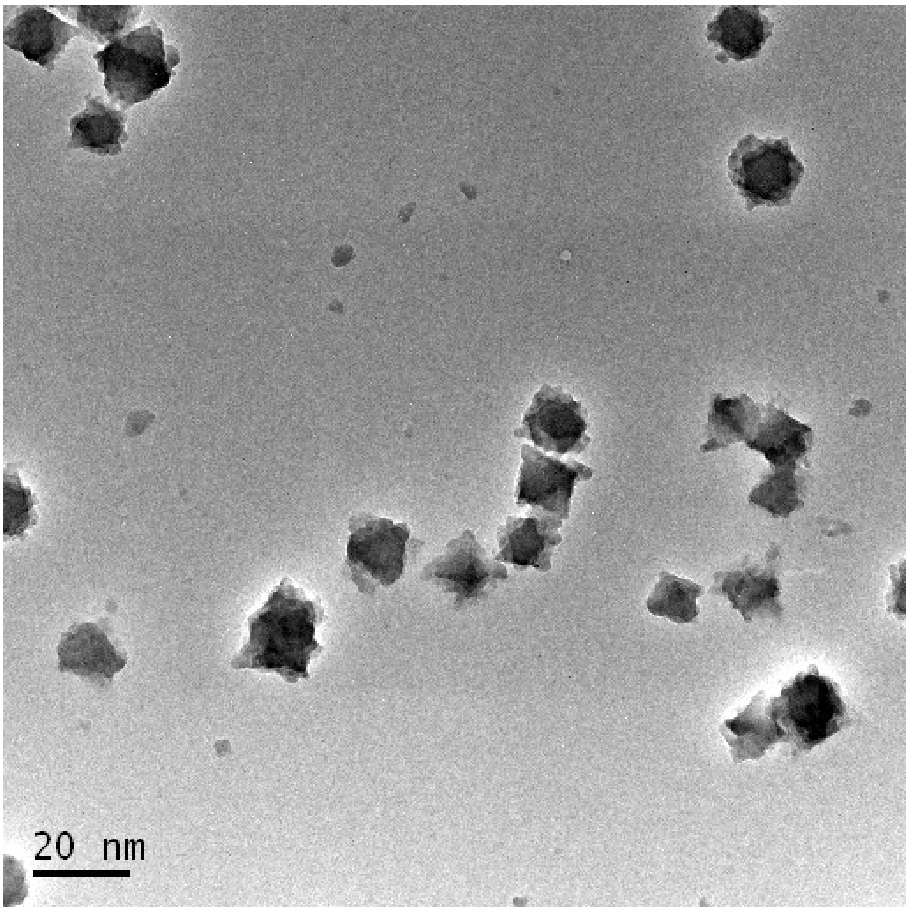

Fig. S9 TEM images of MIL-101 etched by tannic acid.

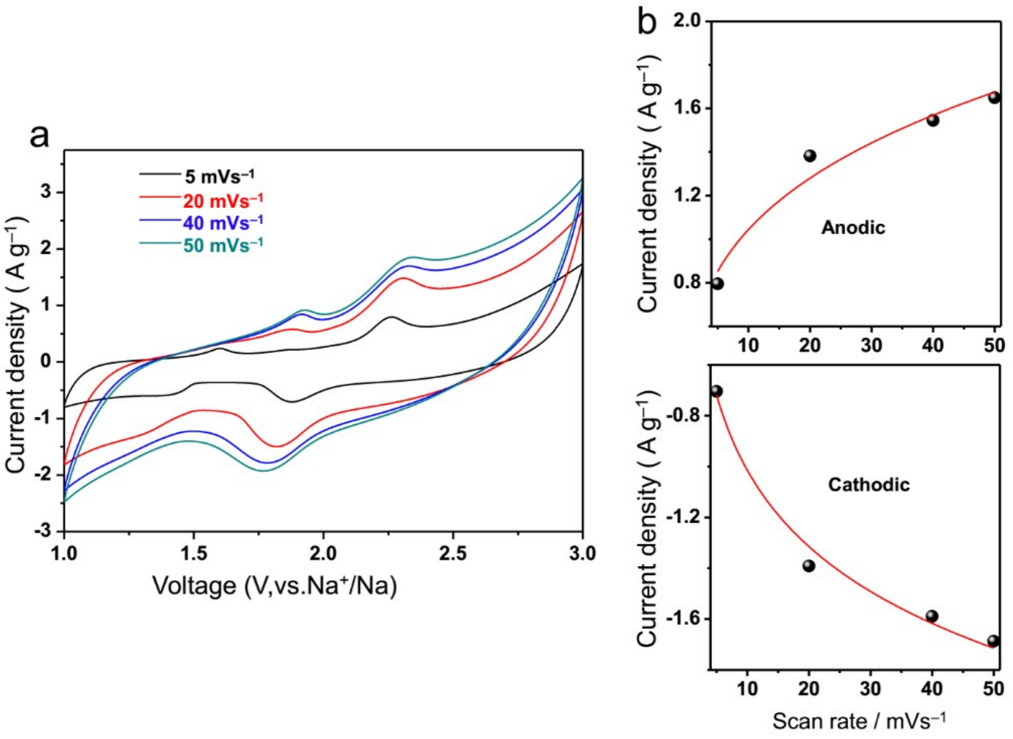

Fig. S10 (a) CV curves of the non-hollow carbon nanoparticles between 1 and 3 V (vs Na^+/Na) at various rate in the range of 5-40 mV·s^{-1}. (b) The dependence of anodic and cathodic current (at 2.0 V) on scanning rate.

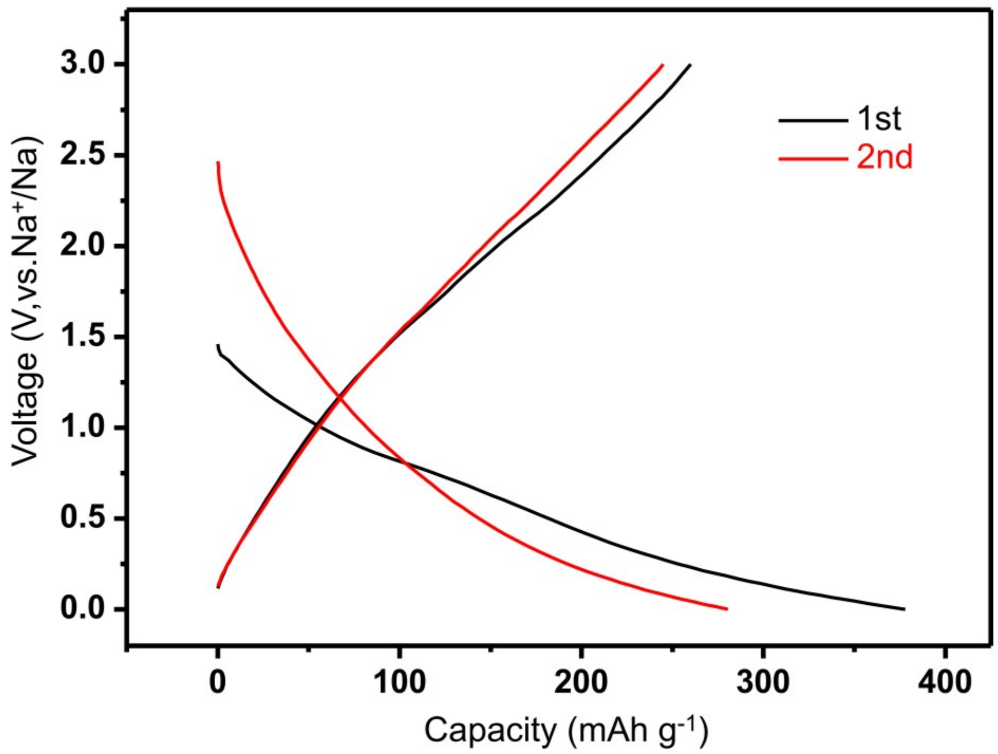

Fig. S11 Galvanostatic charge/discharge curves for the hollow carbon nanobubbles between 0.001 and 3.000 V (vs Na^+/Na) at a current density of 50 mA·g^{-1}.

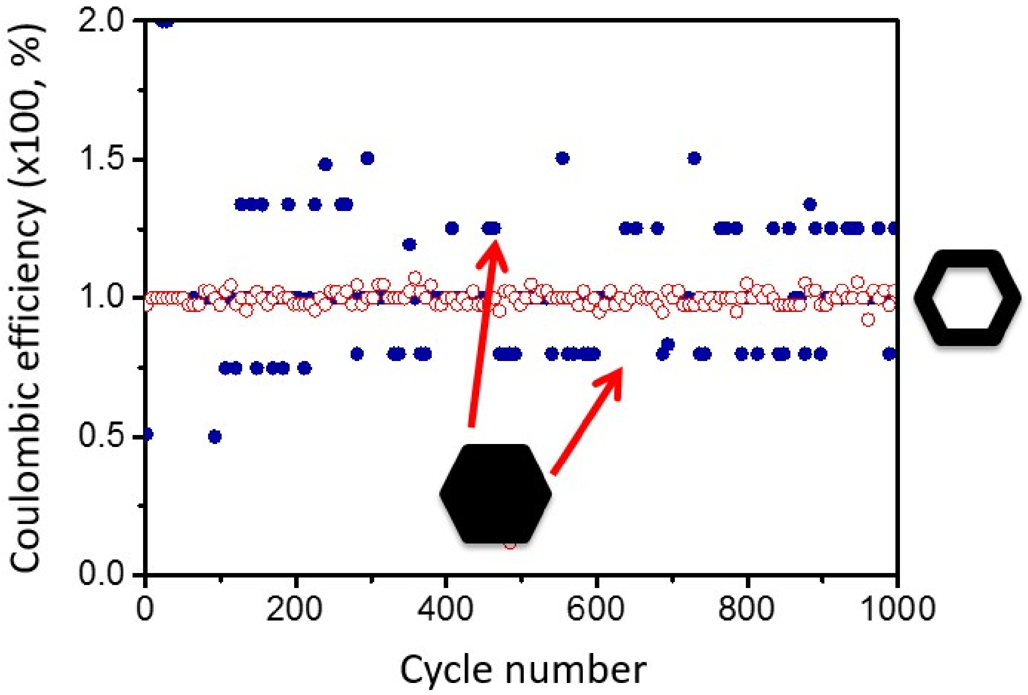

Fig. S12 Coulombic efficiency of the electrodes prepared with non-hollow carbon nanoparticles and hollow carbon nanobubbles cycled at a current density of 10,000 mA·g^{-1}.

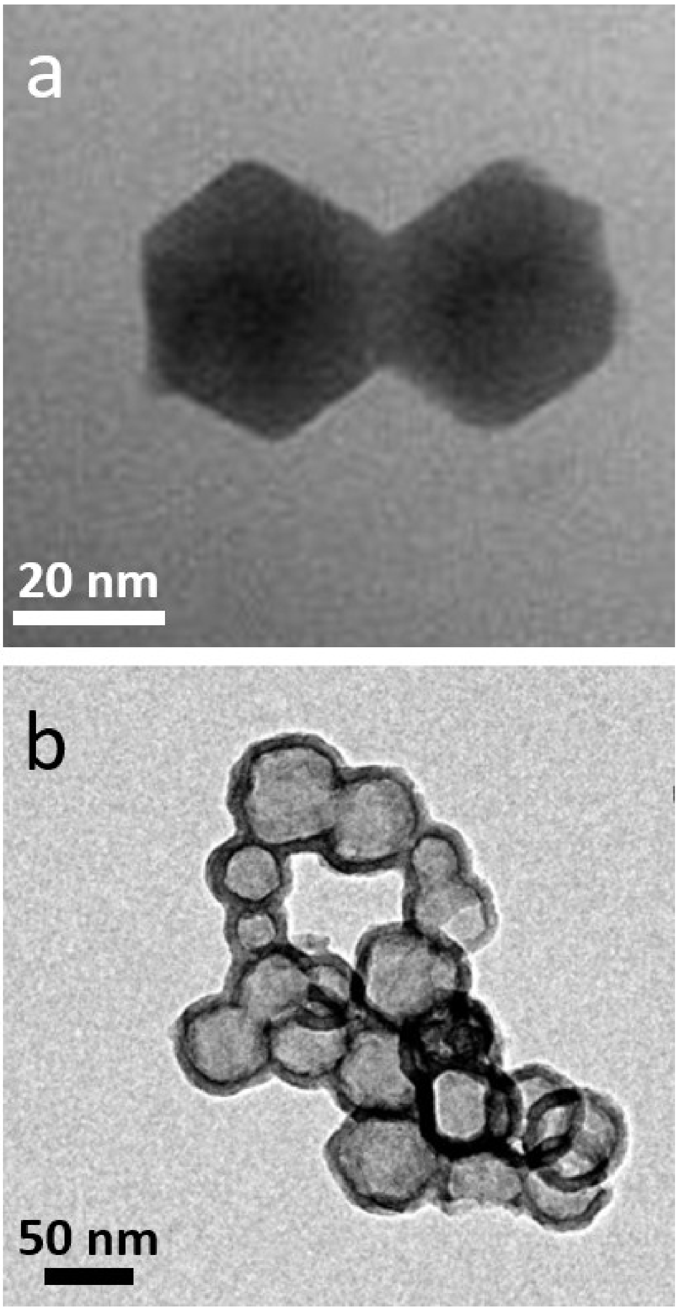

Fig. S13 TEM images of (a) the non-hollow carbon nanoparticles and (b) the hollow carbon nanobubbles after 1,000 charge/discharge cycles at a current density of 10,000 mA g^{-1}.

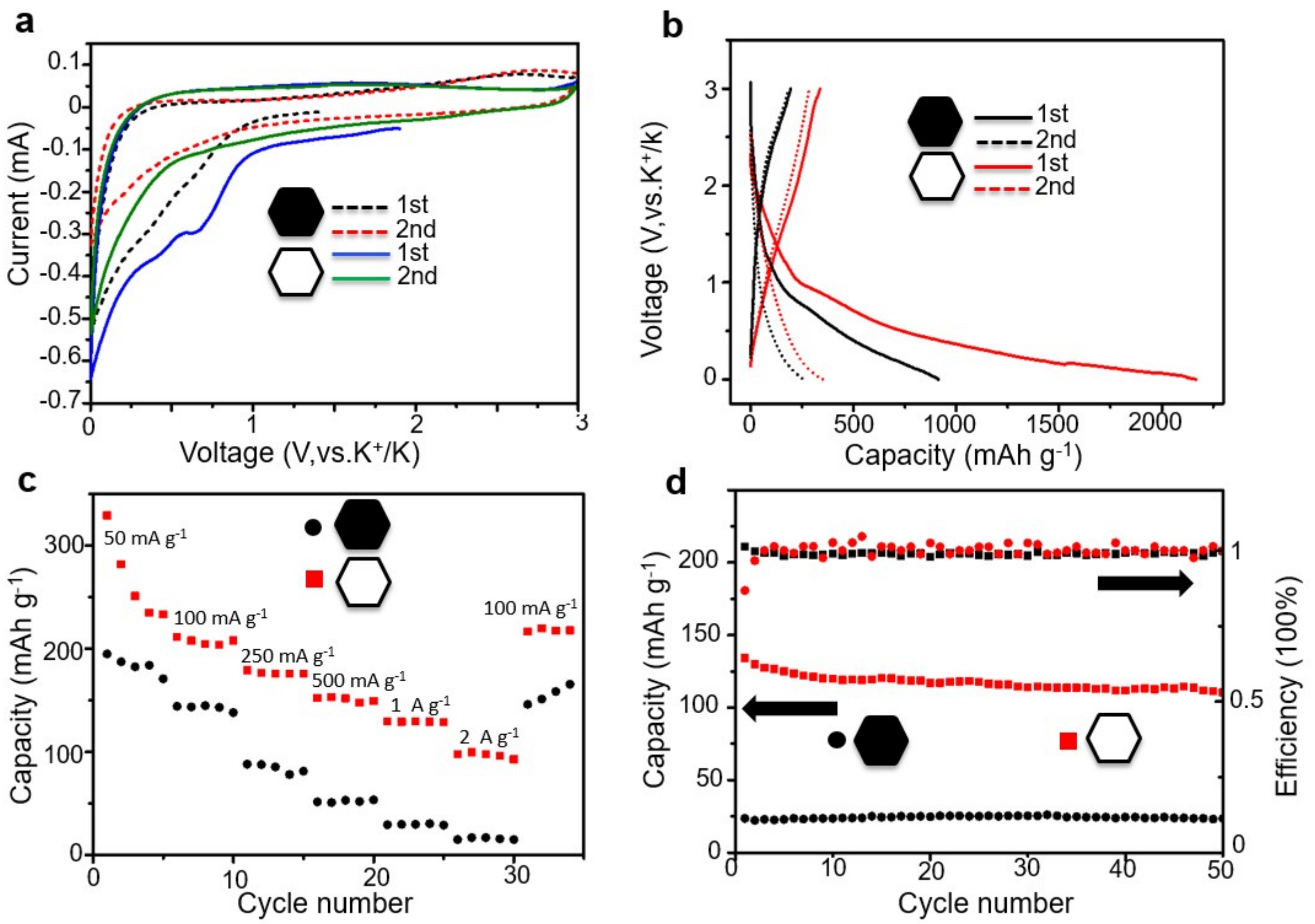

Fig. S14 (a) CV curves for the first 2 cycles of the hollow carbon nanobubbles and non-hollow carbon nanoparticles between 0.001 and 3.000 V (*vs.* K^+/K) at a potential sweep rate of 0.1 mV s^{-1}. (b) Galvanostatic charge/discharge curves for the first 2 cycles between 0.001 and 3.000 V (*vs.* K^+/K) at a current density of 50 mA g^{-1}. (c) Rate performance and (d) cycling performance of the hollow carbon nanobubbles and non-hollow carbon nanoparticles. The electrodes were first activated by discharge/charge at a current density of 50 mA·g^{-1} in the initial cycle before testing at the current density of 1 A·g^{-1}.

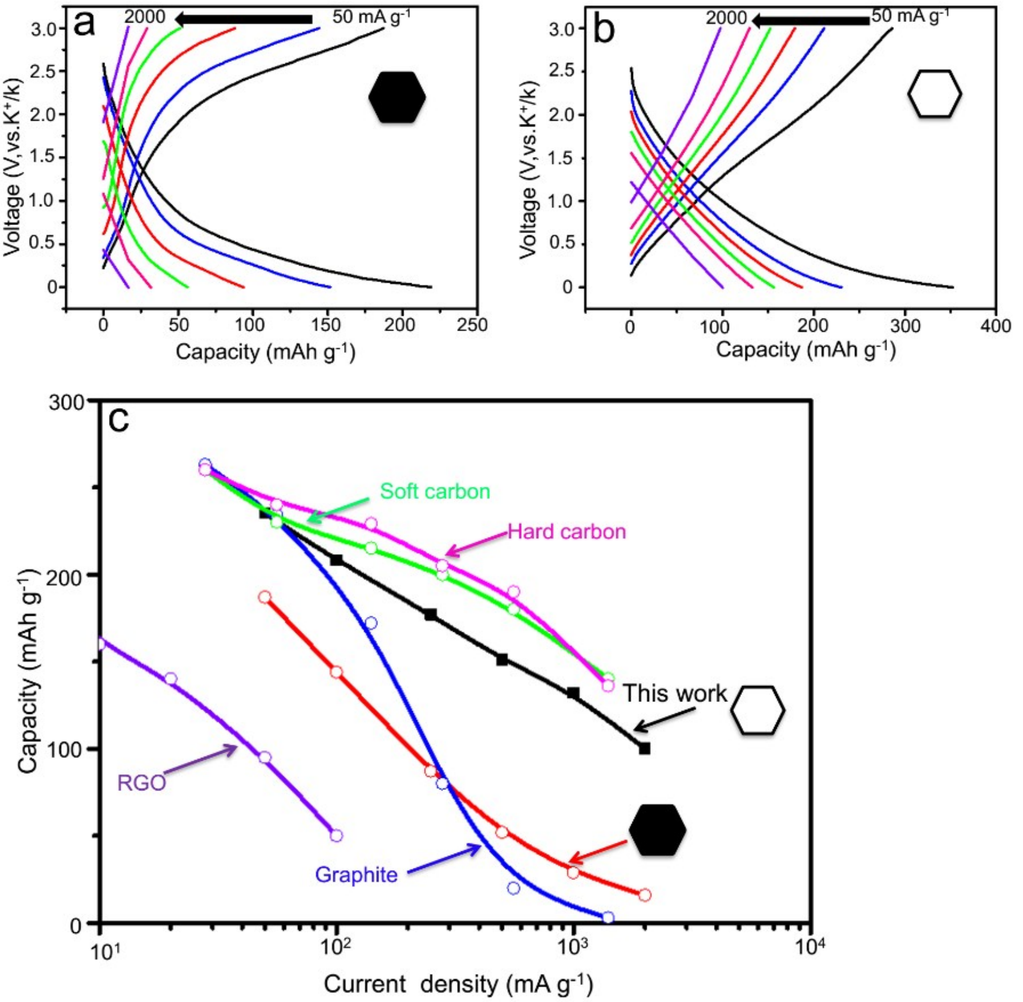

Fig. S15 (a, b) Galvanostatic charge/discharge curves of the non-hollow carbon nanoparticles and the hollow carbon nanobubbles between 0.001 and 3.000 V (*vs*. K^+/K) at various current densities ranging from 50 to 2,000 mA·g^{-1}. (c) Comparison of rate capacity of our carbon materials with other published materials such as hard carbon microspheres[R1], soft carbon[R2], reduced graphene oxide (RGO), and graphite[R3].

R1 Jian, Z., Xing, Z., Bommier, C., Li, Z. & Ji, X. Hard Carbon Microspheres: Potassium-Ion Anode versus Sodium-Ion Anode. *Advanced Energy Materials* **6**, 1501874 (2016).

R2 Jian, Z., Luo, W. & Ji, X. Carbon Electrodes for K-Ion Batteries. *Journal of the American Chemical Society* **137**, 11566-11569 (2015).

R3 Luo, W. *et al.* Potassium Ion Batteries with Graphitic Materials. *Nano Letters* **15**, 7671-7677 (2015).

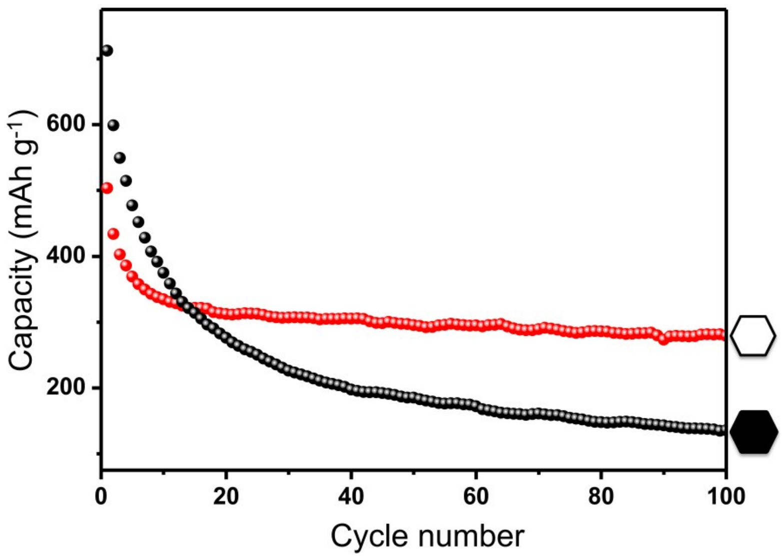

Figure S16. Cycling performance of non-hollow carbon and hollow carbon as cathodes for Li-S battery at a current density of 100 mA·g^{-1}.
